# Survey dataset on the prevalence of childhood maltreatment history among drug addicts in Malaysia

**DOI:** 10.1016/j.dib.2020.105864

**Published:** 2020-06-16

**Authors:** Loy See Mey, Rozainee Khairudin, Tengku Elmi Azlina Tengku Muda, Daniella M Mokhtar, Mohammad Rahim Kamaluddin

**Affiliations:** a Centre for Research in Psychology and Human Well-being, Faculty of Social Sciences and Humanities, Universiti Kebangsaan, Malaysia; b Genius@Pintar National Gifted Centre, Universiti Kebangsaan, Malaysia

**Keywords:** Childhood maltreatment, Childhood trauma questionnaire-short form, Drug addicts, Drug addiction, Malaysia

## Abstract

Studies have consistently shown that childhood maltreatment is a significant risk factor for the development of drug addiction across human lifespan. Yet, little is known about the prevalence of childhood maltreatment history among drug addicts in Malaysia. The dataset presented in this article provides demographic information on 200 drug addicts recruited from two rehabilitation centres in Malaysia, the prevalence of different types of childhood maltreatment history and the correlation between all types of maltreatments. Analyses of the data can provide insights into the prevalence of maltreatment history and development of drug addiction, therefore indispensable for mental health professionals designing appropriate interventions for the drug addicts. The data can also provide baseline data for comparative studies in terms of childhood maltreatment history and drug addiction across different countries.

Specifications Table

**Table:** 

Subject	Social Sciences
Specific subject area	Child abuse, Childhood Maltreatment, Drug Addiction, Developmental Psychology
Type of data	Tables
How data were acquired	Field Survey: Data were collected through the Childhood Trauma Questionnaire Short Form (CTQ-SF) and analyzed via descriptive and inferential statistics using SPSS version 22.0 (IBM Statistical Package for Social Science)
Data format	Raw and descriptive
Parameters for data collection	Participants were 200 drug addicts which were selected using a random sampling technique.
Description of data collection	Participants were recruited from two selected drug rehabilitation centres in Malaysia. Participants were briefed on questionnaire details and data were collected group by group after obtaining the signed consent. The purpose of the study and ethical issues pertaining to the study were clearly explained by the researchers prior to data collection.
Data source location	Institution: Two drug rehabilitation centres under the governance of National Anti-Drugs Agency (NADA) Malaysia City/Town/Region: Jelebu, Negeri Sembilan (2.9984° N, 102.0883° E) & Tiang Dua, Melaka (2.2127° N, 102.3627° E)
Data accessibility	The raw data files are provided in the Data in Brief Dataverse. All other data is within this article.

## Value of the data

• The data provide insights into the prevalence of childhood maltreatment history among drug addicts in Malaysia.
• The data reveal the correlations between different types of childhood maltreatment which further substantiate the coexistence of one and more types of child abuse and neglect in a family.
• The data would be indispensable to researchers and psychologists in designing unique intervention therapies and services for drug addicts entering treatment and rehabilitation.
• The data was collected in Malaysia and specifically concerns Malaysian drug addicts, thus possessing the added value of localised focus and cultural diversity for the incorporation in meta-analyses for comparative studies.
• This dataset allows other researchers to extend statistical analyses about the effects of childhood maltreatment among drug addicts.

## 1 Data description

Childhood maltreatment experiences including physical, emotional, sexual abuses and neglect are well known as a significant risk factors for drug addiction and are common in the life histories of individuals with drug abuse problems [1], [2], [3]. The data cover a set of responses about the childhood maltreatment history obtained from drug addicts from two randomly selected drug rehabilitation centres run by the government of Malaysia. 200 male inpatient drug addicts entering substance abuse treatment were selected through a simple random sampling. An anonymous questionnaire with no identifying information was administered to the participants in a group format of five participants per group. A preliminary briefing session was conducted prior to the questionnaire administration to inform the participants about the research objectives and the contents of the questionnaire as well as ethical measures. The demographic characteristics of participants are shown in Table 1. The mean age of the participants is 33.25 years (SD=7.245) with the youngest and the oldest participants were 20 and 55 years old respectively. The data collection procedures were approved and consented by the National Anti-drugs Agency (NADA) of Malaysia.

**Table 1:** Participant demographic information.

Demographic variables		*n* = 200	Percentage
Age group	20–24	17	8.5
	25–29	62	31
	30–34	43	21.5
	35–39	35	17.5
	40–44	27	13.5
	45–49	13	6.5
	50–55	3	1.5
Ethnicity	Malay	185	92.5
	Chinese	9	4.5
	India	6	3
Marital status	Single	104	52
	Married	73	36.5
	Divorced	20	10
	Widowed	3	1.5
Educational level	Primary	28	14
	PMR/PT3 (Lower secondary school)	53	26.5
	SPM (Upper secondary school)	103	51.5
	STPM/Diploma	8	4
	Bachelor degree	2	1
	Master	1	0.5
	No formal education	5	2.5
Age of first drug use	≤ 13	15	7.5
	14–15	27	13.5
	16–17	34	17
	18–19	32	16
	20–24	53	26.5
	25–29	23	11.5
	30–34	11	5.5
	35–39	5	2.5
Self-reported reasons for	Peer influence	60	30
start taking drugs	Curiosity	64	32
	Problem with parents	8	4
	Pleasure	54	27
	Stress	14	7
Victim of child abuse	Yes	7	3.5
	No	193	96.5

## 2 Experimental design, materials, and methods

### 2.1. Instrument of data collection

The data collection questionnaire consisted of two parts. The first part covers the participants’ demographic information such as age, ethnicity, marital status, educational achievement and age of drug usage initiation. Beside this, self-reported reasons of taking drugs for the very first time and the participants’ perception about their abuse history during childhood with the question “*Have you ever been a victim of child abuse*?” were also included. The responses required are “Yes/No”.

The second part of the instrument consisted of the validated Malay short version of Childhood Trauma Questionnaire (CTQ-SF). This 28-item questionnaire is a self-report screening measure developed by Bernstein et al. for childhood abuse and neglect histories and is the commonly used instrument applied in different languages and contexts [4,5]. CTQ-SF comprised of five sub dimensions/scales or five types of maltreatment: emotional abuse (EA, e.g. “people in my family said hurtful or insulting things to me”), physical abuse (PA, e.g. “I got hit so hard by someone in my family”), sexual abuse (SA, e.g. “someone tried to make me do sexual things or watch sexual things”), emotional neglect (EN, e.g. “my family was a source of strength and support”, reverse coded), and physical neglect (PN, e.g. “I didn't have enough to eat”). Each sub-scale consisted of five items and a sub-dimension with three items namely Minimization/Denial (MD) Scale for detecting denial of abuse are not included in the calculation of CTQ-SF. However, 1 point is given for each item under MD Scale endorsed with a score of 5 while 0 points are given for each item endorsed with a score less than 5. Participants were asked to report on how often they experienced maltreatment during their first 16 years of life on a five-point Likert-scale ranging from 1 “never true” to 5 “very often true”. The sum of each subscale ranges from 5 to 25 with higher scores indicate a higher extent of maltreatment experience. [4], [5], [6].

Overall, the five sub-scale of CTQ-SF which comprised of 25 items as well as three items Minimization/Denial Scale are shown in Table 2. According to the original manual of CTQ-SF, the cut-off scores for each subscale were calculated in order to determine whether participants’ report of childhood maltreatment histories achieved clinically significant abuses or neglect. Bernstein et al. classified the scores on four levels ranging from none/minimal, low, moderate and severe [5]. Subsequently, Walker et al. developed slightly different cut-off criteria to identify the achievement of significant level of traumatic experiences [7]. The overview of both cut-off criteria for five subscales is shown in Table 3.

**Table 2:** CTQ items by scale.

Sub-scales	Items
Emotional abuse	5 (3, 8, 14, 18, 25)
Physical abuse	5 (9, 11, 12, 15, 17)
Sexual abuse	5 (20, 21, 23, 24, 27)
Emotional neglect	5 (5, 7, 13, 19, 28)
Physical neglect	5 (1, 2, 4, 6, 26)
Minimization/Denial	3 (16, 22, 10)

**Table 3:** CTQ cut-off scores according to neglect and abuses.

	Walker et al. [7]	Bernstein and Fink [5]			
		None/minimal	Low	Moderate	Severe
Emotional abuse	≥ 10	≤ 8	9–12	13–15	≥ 16
Physical abuse	≥ 8	≤ 7	8–9	10–12	≥ 13
Sexual abuse	≥ 8	≤ 5	6–7	8–12	≥ 13
Emotional neglect	≥ 15	≤ 9	10–14	15–17	≥ 18
Physical neglect	≥ 8	≤ 7	8–9	10–12	≥ 13

### 2.2. Data presentation

Tables 4 and 5, respectively, show the overview of the prevalence of childhood maltreatment history among the drug addicts according to the cut-off criteria as recommended by Bernstein et al. [5] and Walker et al. [7]. The total of the CTQ-SF reached an average of 42.48 (SD=12.18). Pearson correlation between all five types of maltreatment experiences are presented in Table 6. The highest correlations were found between emotional abuse and physical abuse (*r* = 0.638, *p* ≤ 0.01). Besides, emotional abuse reported for the highest correlation with the total scale (*r* = 0.864, *p* ≤ 0.01) while sexual abuse (*r* = 0.343, *p* ≤ 0.01) reported the least correlation.

**Table 4:** Frequencies of participants achieving cut-off scores in CTQ subscales according to Berstein et al. (*n* = 200).

CTQ subscales	None/minimal		Low		Moderate		High	
	*n*	%	*n*	%	*n*	%	*n*	%
Emotional abuse	76	38	61	30.5	34	17	29	14.5
Physical abuse	124	62	32	16	33	16.5	11	5.5
Sexual abuse	131	65.5	48	24	19	9.5	2	1
Emotional neglect	85	42.5	49	24.5	43	21.5	23	11.5
Physical neglect	138	69	34	17	23	11.5	5	2.5

**Table 5:** Frequencies of participants achieving cut-off scores in CTQ subscales according to Walker et al. (*n* = 200).

CTQ subscales	*n*	%	Mean	SD
Emotional abuse	101	50.5	14.30	3.66
Physical abuse	76	38	10.29	2.15
Sexual abuse	21	10	9.76	1.7
Emotional neglect	66	33	17.33	2.48
Physical neglect	62	31	9.87	2.01

**Table 6:** Pearson correlations among the five CTQ subscales (*n* = 200).

	Total CTQ-SF	Physical abuse	Sexual abuse	Emotional neglect	Physical neglect
Emotional abuse	.864⁎⁎	.638⁎⁎	.202⁎⁎	.604⁎⁎	.373⁎⁎
Physical abuse	.724⁎⁎		.189⁎⁎	.446⁎⁎	.285⁎⁎
Sexual abuse	.343⁎⁎			.144*	.183⁎⁎
Emotional neglect	.853⁎⁎				.547⁎⁎
Physical neglect	.641⁎⁎				

⁎⁎ *p* ≤ 0.01 (2-tailed).

⁎ *p* ≤ 0.05 (2-tailed).

## Declaration of Competing Interest

The authors declare that they have no known competing financial interests or personal relationships which have, or could be perceived to have, influenced the work reported in this article.

## Appendix Supplementary materials

Image, application 1

Image, application 2

Image, application 3
