# Supplementary material for: Survey dataset on the prevalence of childhood maltreatment history among drug addicts in Malaysia
Source: Data Brief. 2020 Jun 16;31:105864. doi: 10.1016/j.dib.2020.105864 (PMC7316995; doi:10.1016/j.dib.2020.105864)
Supplement: Supplementary file 1 [file mmc1.zip › CTQ-SF English verion.docx]

**
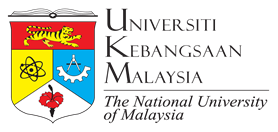
**

**SECTION A**

INSTRUCTIONS: Please make tick mark in check boxes for selection of options and fill in the blanks for specific questions.

1. Gender:

( ) Male ( ) Female

2. Age: ___________ year old

3. Ethnicity:

( ) Malay ( ) Chinese ( ) India ( ) Others, please mention: ________________

4. Education:

( ) Primary school ( ) PMR/PT3 ( ) SPM ( ) STPM

( ) No schooling complete ( ) Bachelor degree ( ) Master/PhD ( ) Others: ___________

5. Marital status:

( ) Single ( ) Married ( ) Divorced ( ) Widowed

6. Are you follow the treatment voluntarily or forced by others?

( ) Voluntary

( ) Forced by others (e.g.,: family, authorities): ____________________________

7. How long have you been following the treatment?_______________________ months

8. What was your age at first time using drugs?___________________ year old

9. Reasons for you to take drugs:

( ) Influence of friends ( ) Curiosity ( ) Problems with parents

( ) For fun ( ) Stress (e.g., family, work, financial):______________________________

( ) Others, please mention: _________________________________________________________

10. Have you been a victim of child abuse? If "yes," go to question 11, if “no”, go to question 12.

( ) Yes, please mention (e.g.,: parents, relatives): __________________ ( ) No

11. If Yes, what kind of abuse did you experience?

( ) Physical (often beaten by parents/siblings/caregivers until injured / bruised)

( ) Verbal / Emotional (always ridiculed / mocked / reprimanded by adults with hurtful words that may lead to low self-esteem and loss of dignity)

( ) Sexual (ever coerced, seduced, threatened by adults for sexual activity)

( ) Neglected by parents/caregivers (physical/emotional), please indicate: ________________________

12. What is your motivation for treatment at Cure & Care Rehabilitation Centre?

( ) Very high ( ) High ( ) Medium ( ) Low

**SECTION B**

Directions: These questions ask about some of your experiences growing up as a child and a teenager. For each question, indicate and circle the degree that best describes how you feel. Although some of these questions are of a personal nature, please try to answer as honestly as you can. Your answers will be kept confidential.

| 1 | 2 | 3 | 4 | 5 |
| --- | --- | --- | --- | --- |
| Never true | Rarely true | Sometimes true | Often true | Very often true |

|  | **Item** | **Scale** | | | | |
| --- | --- | --- | --- | --- | --- | --- |
|  | **When I was growing up, . . .** | | | | | |
| 1 | **(PN)** I didn't have enough to eat. | 1 | 2 | 3 | 4 | 5 |
| 2 | **(PN)** I knew that there was someone to take care of me and protect me. | 1 | 2 | 3 | 4 | 5 |
| 3 | **(EA)** People in my family called me things like "stupid", "lazy", or "ugly". | 1 | 2 | 3 | 4 | 5 |
| 4 | **(PN)** My parents were too drunk or high to take care of the family. | 1 | 2 | 3 | 4 | 5 |
| 5 | **(EN)** There was someone in my family who helped me feel important or special. | 1 | 2 | 3 | 4 | 5 |
| 6 | **(PN)** I had to wear dirty clothes. | 1 | 2 | 3 | 4 | 5 |
| 7 | **(EN)** I felt loved. | 1 | 2 | 3 | 4 | 5 |
| 8 | **(EA)** I thought that my parents wished I had never been born. | 1 | 2 | 3 | 4 | 5 |
| 9 | **(PA)** I got hit so hard by someone in my family that I had to see a doctor or go to the hospital. | 1 | 2 | 3 | 4 | 5 |
| 10 | **(MD)** There was nothing I wanted to change about my family. | 1 | 2 | 3 | 4 | 5 |
| 11 | **(PA)** People in my family hit me so hard that it left me with bruises or marks. | 1 | 2 | 3 | 4 | 5 |
| 12 | **(PA)** I was punished with a belt, a board, a cord (or some other hard object). | 1 | 2 | 3 | 4 | 5 |
| 13 | **(EN)** People in my family looked out for each other. | 1 | 2 | 3 | 4 | 5 |
| 14 | **(EA)** People in my family said hurtful or insulting things to me. | 1 | 2 | 3 | 4 | 5 |
| 15 | **(PA)** I believe that I was physically abused. | 1 | 2 | 3 | 4 | 5 |
| 16 | **(MD)** I had the perfect childhood. | 1 | 2 | 3 | 4 | 5 |
| 17 | **(PA)** I got hit or beaten so badly that it was noticed by someone like a friend, teacher, neighbor, or doctor. | 1 | 2 | 3 | 4 | 5 |
| 18 | **(EA)** Someone in my family hated me. | 1 | 2 | 3 | 4 | 5 |
| 19 | **(EN)** People in my family felt close to each other. | 1 | 2 | 3 | 4 | 5 |
| 20 | **(SA)** Someone tried to touch me in a sexual way or tried to make me touch them. | 1 | 2 | 3 | 4 | 5 |
| 21 | **(SA)** Someone threatened to hurt me or tell lies about me unless I did something sexual with them. | 1 | 2 | 3 | 4 | 5 |
| 22 | **(MD)** I had the best family in the world. | 1 | 2 | 3 | 4 | 5 |
| 23 | **(SA)** Someone tried to make me do sexual things or watch sexual things. | 1 | 2 | 3 | 4 | 5 |
| 24 | **(SA)** Someone molested me (took advantage of me sexually). | 1 | 2 | 3 | 4 | 5 |
| 25 | **(EA)** I believe that I was emotionally abused. | 1 | 2 | 3 | 4 | 5 |
| 26 | **(PN)** There was someone to take me to the doctor if I needed it. | 1 | 2 | 3 | 4 | 5 |
| 27 | **(SA)** I believe that I was sexually abused. | 1 | 2 | 3 | 4 | 5 |
| 28 | **(EN)** My family was a source of strength and support. | 1 | 2 | 3 | 4 | 5 |

**THANK YOU ☺**

**Childhood Trauma Questionnaire Items by Scale**

| **Sub-scales** | **Abbreviation** | **Items** |
| --- | --- | --- |
| Emotional abuse | EA | 5 (3, 8, 14, 18, 25) |
| Physical abuse | PA | 5 (9, 11, 12, 15, 17) |
| Sexual abuse | SA | 5 (20, 21, 23, 24, 27) |
| Emotional neglect | EN | 5 (5, 7, 13, 19, 28) |
| Physical neglect | PN | 5 (1, 2, 4, 6, 26) |
| Minimization/Denial | MD | 3 (10, 16, 22) |
