# Supplementary material for: Survey dataset on the prevalence of childhood maltreatment history among drug addicts in Malaysia
Source: Data Brief. 2020 Jun 16;31:105864. doi: 10.1016/j.dib.2020.105864 (PMC7316995; doi:10.1016/j.dib.2020.105864)
Supplement: Supplementary file 1 [file mmc1.zip › CTQ-SF Malay version.docx]

**Kod:**

**SOAL SELIDIK KAJIAN**

**
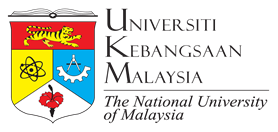
**

**BAHAGIAN A: MAKLUMAT LATAR BELAKANG**

**ARAHAN:** Sila berikan maklumat diri anda dengan tepat dan ikhlas. Tandakan (√) pada petak yang disediakan dan isi tempat kosong bagi soalan-soalan tertentu.

1. Jantina:

( ) Lelaki ( ) Perempuan

2. Umur: ___________ tahun

3. Bangsa:

( ) Melayu ( ) Cina ( ) India ( ) Lain-lain, nyatakan: ________________

4. Tahap pendidikan tertinggi:

( ) Sekolah rendah ( ) PMR/PT3 ( ) SPM ( ) STPM

( ) Tidak bersekolah ( ) Ijazah sarjana muda ( ) Master/PhD ( ) Lain-lain, nyatakan: ___________

5. Status perkahwinan:

( ) Bujang ( ) Berkahwin ( ) Bercerai ( ) Pasangan meninggal

6. Adakah anda rela mengikuti rawatan atau dipaksa oleh orang lain?

( ) Sukarela

( ) Dipaksa, nyatakan (cth: ahli keluarga/pihak berkuasa dll): ____________________________

7. Berapa lama anda telah mengikuti rawatan pemulihan? _______________________ bulan

8. Apakah umur anda semasa kali **PERTAMA** menggunakan dadah? ___________________ tahun

9. Punca anda mengambil dadah:

( ) Pengaruh kawan ( ) Perasaan ingin tahu ( ) Masalah dengan ibu/bapa

( ) Keseronokan ( ) Tekanan, nyatakan (keluarga, kerja, kewangan):____________

( ) Lain-lain, nyatakan: _________________________________________________________

10. Pernah anda menjadi mangsa dera semasa zaman kanak-kanak? Jika “ya”, pergi ke soalan 11, jika tidak, terus ke soalan 12.

( ) Ya, nyatakan (cth: ibu/bapa/saudara dll): __________________ ( ) Tidak

11. Jika pernah, apakah jenis penderaan yang anda alami?

( ) Fizikal (selalu dipukul oleh ibu/bapa/saudara/penjaga sehingga cedera/lebam)

( ) Verbal/Emosional (selalu diejek/dihina/ditegur oleh dewasa dengan ungkapan kasar dan kata-kata yang menyakitkan sehingga mendatangkan perasaan rendah diri dan hilang maruah)

( ) Seksual (pernah dipaksa, diperdaya, diugut oleh orang dewasa untuk melakukan aktiviti seksual)

( ) Pengabaian oleh ibu/bapa/penjaga (fizikal/emosi), nyatakan: ________________________________

12. Apakah tahap motivasi anda mengikuti rawatan di CCRC?

( ) Sangat tinggi ( ) Tinggi ( ) Sederhana ( ) Rendah

**BAHAGIAN B**

**Arahan**: Soalan-soalan berikut bertanya tentang pengalaman anda sebagai seorang kanak-kanak dan remaja sepanjang proses pertumbuhan dan perkembangan. Dengan menggunakan skala di bawah sebagai panduan, sila **bulatkan satu nombor** yang paling sesuai menggambarkan perasaan anda.

| 1 | 2 | 3 | 4 | 5 |
| --- | --- | --- | --- | --- |
| Langsung tidak benar | Jarang benar | Kadang-kadang benar | Selalu benar | Sangat benar |

|  | **Item** | **Skala** | | | | |
| --- | --- | --- | --- | --- | --- | --- |
|  | **Sepanjang proses pembesaran/perkembangan saya dari kanak-kanak hingga remaja…** | | | | | |
| 1 | **(PN)** Saya tidak cukup makan. | 1 | 2 | 3 | 4 | 5 |
| 2 | **(PN)** Saya tahu ada orang yang menjaga dan melindungi saya. | 1 | 2 | 3 | 4 | 5 |
| 3 | **(EA)** Ahli keluarga saya pernah menggelar saya dengan panggilan seperti "bodoh", "malas", atau "hodoh". | 1 | 2 | 3 | 4 | 5 |
| 4 | **(PN)** Ibu/bapa saya selalunya terlalu mabuk sehingga tidak mampu menjaga keluarga. | 1 | 2 | 3 | 4 | 5 |
| 5 | **(EN)** Ada ahli keluarga saya yang membuatkan saya merasa penting atau istimewa. | 1 | 2 | 3 | 4 | 5 |
| 6 | **(PN)** Saya terpaksa memakai pakaian kotor. | 1 | 2 | 3 | 4 | 5 |
| 7 | **(EN)** Saya berasa disayangi. | 1 | 2 | 3 | 4 | 5 |
| 8 | **(EA)** Saya merasakan ibu bapa saya berharap saya tidak pernah dilahirkan. | 1 | 2 | 3 | 4 | 5 |
| 9 | **(PA)** Saya pernah dipukul dengan kuat oleh ahli keluarga sehingga perlu berjumpa doktor. | 1 | 2 | 3 | 4 | 5 |
| 10 | **(MD)** Tiada apa yang saya ingin ubah mengenai keluarga saya. | 1 | 2 | 3 | 4 | 5 |
| 11 | **(PA)** Ada ahli keluarga saya pernah memukul saya dengan kuat sehingga meninggalkan lebam atau tanda. | 1 | 2 | 3 | 4 | 5 |
| 12 | **(PA)** Saya pernah dipukul dengan tali pinggang, kayu, tali (atau objek keras yang lain). | 1 | 2 | 3 | 4 | 5 |
| 13 | **(EN)** Ahli keluarga saya saling menjaga dan membantu antara satu sama lain. | 1 | 2 | 3 | 4 | 5 |
| 14 | **(EA)** Ahli keluarga saya pernah menghina atau mengatakan sesuatu yang menyakitkan hati saya. | 1 | 2 | 3 | 4 | 5 |
| 15 | **(PA)** Saya percaya bahawa saya telah didera secara fizikal. | 1 | 2 | 3 | 4 | 5 |
| 16 | **(MD)** Saya melalui zaman kanak-kanak yang sangat baik dan sempurna. | 1 | 2 | 3 | 4 | 5 |
| 17 | **(PA)** Saya pernah dipukul dengan teruk sehingga mendapat perhatian orang lain seperti kawan, saudara-mara atau guru. | 1 | 2 | 3 | 4 | 5 |
| 18 | **(EA)** Ada ahli keluarga saya yang benci saya. | 1 | 2 | 3 | 4 | 5 |
| 19 | **(EN)** Hubungan ahli-ahli keluarga saya adalah rapat. | 1 | 2 | 3 | 4 | 5 |
| 20 | **(SA)** Ada orang yang cuba menyentuh saya secara seksual atau cuba membuatkan saya menyentuh mereka. | 1 | 2 | 3 | 4 | 5 |
| 21 | **(SA)** Ada orang pernah mengancam untuk menyakitkan saya atau fitnah tentang saya kecuali saya melakukan aktiviti seksual dengan mereka. | 1 | 2 | 3 | 4 | 5 |
| 22 | **(MD)** Saya mempunyai keluarga yang terbaik di dunia. | 1 | 2 | 3 | 4 | 5 |
| 23 | **(SA)** Ada orang cuba membuatkan saya menonton perkara lucah. | 1 | 2 | 3 | 4 | 5 |
| 24 | **(SA)** Ada orang pernah mencabul saya (mengambil kesempatan secara seksual terhadap saya). | 1 | 2 | 3 | 4 | 5 |
| 25 | **(EA)** Saya percaya bahawa saya telah didera secara emosi. | 1 | 2 | 3 | 4 | 5 |
| 26 | **(PN)** Ada orang yang boleh membawa saya berjumpa doktor jika saya memerlukannya. | 1 | 2 | 3 | 4 | 5 |
| 27 | **(SA)** Saya percaya bahawa saya telah didera secara seksual. | 1 | 2 | 3 | 4 | 5 |
| 28 | **(EN)** Keluarga saya merupakan sumber kekuatan dan sokongan bagi diri saya. | 1 | 2 | 3 | 4 | 5 |

**SEKIAN TERIMA KASIH ☺**

**Soal selidik trauma zaman kanak-kanak berdasarkan sub-skala**

| **Sub-Skala** | **Singkatan** | **Item** |
| --- | --- | --- |
| Penderaan emosi | EA | 5 (3, 8, 14, 18, 25) |
| Penderaan fizikal | PA | 5 (9, 11, 12, 15, 17) |
| Penderaan seksual | SA | 5 (20, 21, 23, 24, 27) |
| Pengabaian emosi | EN | 5 (5, 7, 13, 19, 28) |
| Pengabaian fizikal | PN | 5 (1, 2, 4, 6, 26) |
| *Minimization/Denial* | MD | 3 (10, 16, 22) |
